# Gamification Boosts Physical Activity in Disparate Populations of Cancer Survivors

**DOI:** 10.1016/j.jaccao.2025.12.010

**Published:** 2026-04-21

**Authors:** Sanglin Zhao

I read with great interest the report on the ALLSTAR (RCT of Strategies to Augment Physical Activity in Black and Hispanic Breast and Prostate Cancer Survivors) trial by Fanaroff et al,^1^ which demonstrated that a behaviorally designed gamification intervention effectively increased physical activity among Black and Hispanic survivors of breast and prostate cancer with elevated cardiovascular risk. This work addresses a critical gap, as this population faces a disproportionate cardiovascular burden and barriers to physical activity.

The success of the intervention lies in its integration of behavioral economic principles, including precommitment, loss framing, and social responsibility, delivered remotely via text messages and wearable devices. Gamification participants increased their daily steps by an average of 759 during the 24-week intervention period, representing a 16% increase from baseline. They also maintained moderate to vigorous physical activity during the follow-up period (an increase of 11 minutes per week; *P* = 0.048). This aligns with previous evidence that gamification outperforms standard care in enhancing physical activity among high-risk populations.^2^^,^^3^

A key advantage is scalability: fully automated delivery eliminates the need for clinical personnel, reducing barriers such as access to fitness facilities or transportation, which is critically important for overcoming disparities.^4^^,^^5^ Personalized goal setting also addresses the impracticality of one-size-fits-all guidelines, as only 2.6% of participants met the moderate to vigorous physical activity recommendations at baseline. This intervention can also be easily integrated into the health care systems.

Although I commend the investigators for prioritizing underserved populations and providing sustainable solutions, I note that the trial excluded non-English and non-Spanish speakers and had limited representation of survivors of prostate cancer. Future research should explore long-term cardiovascular outcomes and adaptation to other cancer types. However, this trial offers a viable model for reducing health disparities.
